# A Comparison of Variant Calling Pipelines Using Genome in a Bottle as a Reference

**DOI:** 10.1155/2015/456479

**Published:** 2015-10-11

**Authors:** Adam Cornish, Chittibabu Guda

**Affiliations:** ^1^Department of Genetics, Cell Biology and Anatomy, University of Nebraska Medical Center, Omaha, NE 68198, USA; ^2^Bioinformatics and Systems Biology Core, University of Nebraska Medical Center, Omaha, NE 68198, USA; ^3^Department of Biochemistry and Molecular Biology, University of Nebraska Medical Center, Omaha, NE 68198, USA; ^4^Fred and Pamela Buffet Cancer Center, University of Nebraska Medical Center, Omaha, NE 68198, USA; ^5^Eppley Institute for Research in Cancer and Allied Diseases, University of Nebraska Medical Center, Omaha, NE 68198, USA

## Abstract

High-throughput sequencing, especially of exomes, is a popular diagnostic tool, but it is difficult to determine which tools are the best at analyzing this data. In this study, we use the NIST Genome in a Bottle results as a novel resource for validation of our exome analysis pipeline. We use six different aligners and five different variant callers to determine which pipeline, of the 30 total, performs the best on a human exome that was used to help generate the list of variants detected by the Genome in a Bottle Consortium. Of these 30 pipelines, we found that Novoalign in conjunction with GATK UnifiedGenotyper exhibited the highest sensitivity while maintaining a low number of false positives for SNVs. However, it is apparent that indels are still difficult for any pipeline to handle with none of the tools achieving an average sensitivity higher than 33% or a Positive Predictive Value (PPV) higher than 53%. Lastly, as expected, it was found that aligners can play as vital a role in variant detection as variant callers themselves.

## 1. Background

In the past few years there have been many advances made to high-throughput sequencing technologies. Due to these advances, it is now possible to detect a great number of potential disease-causing variants [1], and, in a few cases, next generation sequencing (NGS) data has even been used for diagnostic purposes [2–4]. This is partially due to the developments in sequencing technologies over the past few years but also due to the number of improvements made to the various bioinformatic tools used to analyze the mountains of data produced by NGS instruments [5].

When searching for mutations in a patient, a typical workflow is to sequence their exome with an Illumina sequencer, align the raw data to the human reference genome, and then identify single nucleotide variants (SNVs) or short insertions and deletions (indels) that could possibly cause or influence the phenotype of interest [6]. While this is fairly straightforward, deciding on the best tools to use at each stage of the analysis pipeline is not. There are a large number of tools that are used in various intermediate steps, but the two most important steps in the entire process are aligning the raw reads to the genome and then searching for variants (i.e., SNVs and indels) [7]. In this study, we aim to help today's bioinformatician by elucidating the correct combination of short read alignment tool and variant calling tool for processing exome sequencing data produced by NGS instruments.

A number of these studies have been performed in the past, but they all had drawbacks of some form or another. Ideally one should have a list of every known variant contained in a sample so that when a pipeline of analysis tools is run, you can test it to know with certainty that it is performing correctly. However, in the past no such list existed, so validation had to be performed by less complete methods. In some instances, validation was performed by generating simulated data so as to create a set of known true positives (TP) and true negatives (TN) [8–10]. While this conveniently provides a list of every TP and TN in the dataset, it does a poor job of accurately representing biology. Other methods of validating variant calling pipelines include using genotyping arrays or Sanger sequencing to obtain a list of TPs and false positives (FP) [11]. These have the upside of providing biologically validated results, but they also have the downside of not being comprehensive due to the limited number of spots on genotyping arrays and the prohibitive cost of Sanger validation when performed thousands of times. Lastly, none of these studies aimed at looking at the effect the short read aligner had on variant calling. Consequently, the upstream effect of aligner performance could not be assessed independently.

In this study, we have the advantage of a list of variants for an anonymous female from Utah (subject ID: NA12878, originally sequenced for the 1000 Genomes project [12]) that was experimentally validated by the NIST-led Genome in a Bottle (GiaB) Consortium. This list of variants was created by integrating 14 different datasets from five different sequencers, and it allows us to validate any list of variants generated by our exome analysis pipelines [7]. The novelty of this work is to validate the right combination of aligners and variant callers against a comprehensive and experimentally determined variant dataset: NIST-GiaB.

To perform our analysis we will be using one of the exome datasets originally used to create the NIST-GiaB list. We chose only one of the original Illumina TruSeq-generated exomes because we wanted to provide a standard use case scenario for someone who wishes to perform NGS analysis, and while whole genome sequencing is continuing to drop in price, exome sequencing is still a popular and viable alternative [1]. It is also important to note that, per Bamshad et al., currently the expected number of SNVs per European-American exome is 20,283 ± 523 [13]. Despite this, the total number of SNVs found in the NIST-GiaB list with the potential to exist in TruSeq exome dataset was 34,886, which is significantly higher than expected. This is likely due to the fact that while the exome kit was used to generate NIST-GiaB data it was also supplemented by whole genome sequencing.

Lastly, we considered a large number of aligners [14–21] and variant callers [22–29] but ultimately chose the 11 tools based on prevalence, popularity, and relevancy to our dataset (e.g., SNVMix, VarScan2, and MuTect were not used as they are intended for use on tumor-derived samples). Our analysis itself involves comparing six aligners (Bowtie2 [14], BWA sampe [15], BWA mem [16], CUSHAW3 [17], MOSAIK [18], and Novoalign) and five variant callers (FreeBayes [22], GATK HaplotypeCaller, GATK UnifiedGenotyper [23], SAMtools mpileup [24], and SNPSVM [25]). In this study we also try to determine how much of an effect, if any, the aligner has on variant calling and which aligners perform best when using a normal Illumina exome sample. To our knowledge, this is the first report which validates all possible combinations (total of 30 pipelines) of a wide array of aligners and variant callers.

## 2. Methods

### 2.1. Datasets

Human reference genome hg19 was downloaded from the UCSC browser (http://hgdownload.soe.ucsc.edu/goldenPath/hg19/chromosomes/) and was used to perform the alignments. The human exome, SRR098401, was downloaded from the Sequence Read Archive (SRA) (http://www.ncbi.nlm.nih.gov/sra). For annotation and calibration purposes, dbSNP137 without sites after version 129, HapMap 3.3, Human Omni 2.5 BeadChip, and Mills and 1000 G gold standard indel set lists were used (all from ftp://ftp.broadinstitute.org/distribution/gsa/gatk_resources.tgz).

### 2.2. The Pipeline

**Figure 1:**
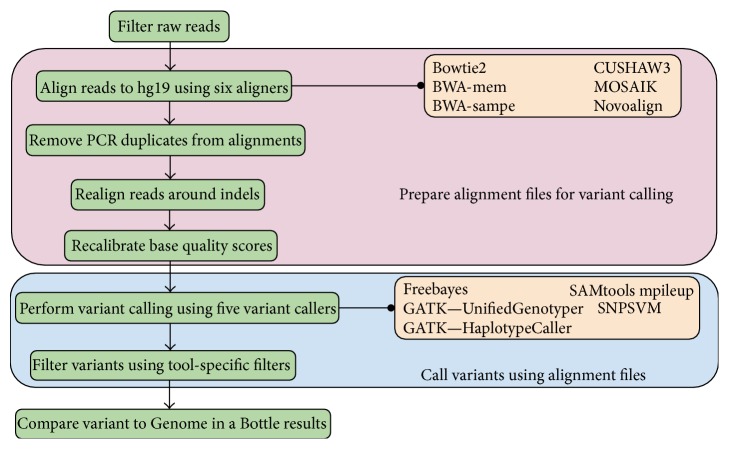
Schematic of the data analysis pipeline used. To ensure that the highest quality alignments are created, reads are first filtered and then aligned to the human reference genome, hg19. Next, PCR duplicates are removed, reads are aligned around putative indels, and base quality scores are recalibrated. Finally, variants are called and validated against the NIST-GiaB list of variants.

Figure 1 shows the workflow used in this study, which is similar to the one outlined in the Best Practices guide produced by The Broad Institute [30]. This involves a number of steps to ensure that the alignment files produced are of the highest quality as well as several more to guarantee the variants are called correctly. First, raw reads were aligned to hg19, and then PCR duplicates were removed from the alignment. Next, to help with indel identification later in the pipeline, read realignment was performed around indels. The last step of alignment processing was to perform a base quality score recalibration step, which helps to ameliorate the inherent bias and inaccuracies of scores issued by sequencers. Unfortunately, despite these steps, the alignment rate of each aligner was significantly lower than expected, so to offset this, the fastx toolkit was used to filter out low quality reads (Table 1). Low quality reads were defined as those reads that had at least half of their quality scores below 30. Following alignment processing, variant calling and variant filtering were performed.

The six tools used to generate alignments were Bowtie2, BWA mem, BWA sampe, CUSHAW3, MOSAIK, and Novoalign, and the five tools used to generate variants were FreeBayes, GATK HaplotypeCaller, GATK UnifiedGenotyper, SAMtools mpileup, and SNPSVM, as can be seen in Table 2.

### 2.3. Filtering

Raw data was acquired from the SRA (SRR098401), split with fastq-dump, and filtered using the fastx toolkit. Specifically, fastq-dump used the  –split_files and  –split_spot flags, and fastq_quality_filter was run with the following arguments: -Q 33 -q 30 -p 50. Then reads were properly paired with a custom script.

### 2.4. Aligning

Aligners used default arguments except when a threads argument was used where available. The commands used are as follows.

#### 2.4.1. Bowtie2

1. bowtie2 -p 10 -x $INDEX -1 raw_data/read_1_filtered.fastq -2 raw_data/read_2_filtered.fastq -S alignments/NA12878.bt2.sam

#### 2.4.2. BWA Sampe

1. bwa aln -t 10 genome/hg19.fa raw_data/read_1_filtered.fastq  >  alignments/NA12878.R1.sai
2. bwa aln -t 10 genome/hg19.fa raw_data/read_2_filtered.fastq  >  alignments/NA12878.R2.sai
3. bwa sampe genome/hg19.fa alignments/NA12878.R1.sai alignments/NA12878.R2.sai raw_data/read_1_filtered.fastq raw_data/read_2_filtered.fastq >  alignments/NA12878.bwa-sampe.sam

#### 2.4.3. BWA Mem

1. bwa mem -t 10 genome/hg19.fa raw_data/read_1_filtered.fastq raw_data/read_2_filtered.fastq  >  alignments/NA12878.bwa-mem.sam

#### 2.4.4. CUSHAW3

1. cushaw3 align -r $INDEX -t 10 -o alignments/NA12878.CUSHAW3.sam -q raw_data/read_1_filtered.fastq raw_data/read_2_filtered.fastq

#### 2.4.5. MOSAIK

1. MosaikBuild -q raw_data/read_1_filtered.fastq -q2 raw_data/read_2_filtered.fastq -st illumina -outalignments/NA12878.MOSAIK.mkb
2. MosaikAligner -in alignments/NA12878.MOSAIK.mkb -out alignments/NA12878.MOSAIK -p 10 -ia genome/hg19.dat -j genome/hg19_15 -annpe tools/MOSAIK/src/networkFile/2.1.78.pe.ann -annse tools/MOSAIK/src/networkFile/2.1.78.se.ann

#### 2.4.6. Novoalign

1. novoalign -d $INDEX -f raw_data/read_1_filtered.fastq raw_data/read_2_filtered.fastq -o SAM -c 10  > alignments/NA12878.novoalign.sam

### 2.5. Alignment Depth of Coverage Calculation

To ensure proper depth of coverage calculation, the Picard Tools module CalculateHsMetrics was used with the following arguments:

1. java -jar CalculateHsMetrics.jar I=NA12878.ALN.BQSR.bam O=ALN.O.log R=genome/hg19.fa TI=genome/truseq_exome.bed BI=genome/truseq_exome.bed VALIDATION_STRINGENCY=SILENT PER_TARGET_COVERAGE=ALN.ptc.bed

It is important to note that the TruSeq exome bed file must have the header from the SAM alignment file prepended to it for this module to function. Further, column 6 must be moved to column 4, and column 5 needs to be removed from the TruSeq bed file.

### 2.6. Alignment File Processing

Processing the alignment files (SAM/BAM files) required the following steps for all aligners:

(1) SAM to BAM conversion with SAMtools view:
  (a) samtools view -bS alignments/NA12878.ALN.sam -o alignments/NA12878.ALN.bam
(2) BAM file sorting using the Picard Tools module, SortSam:
  (a) java -jar bin/SortSam.jar VALIDATION_STRINGENCY=SILENT I=alignments/NA12878.ALN.bam OUTPUT=alignments/NA12878.ALN.sorted.bam SORT_ORDER=coordinate
(3) PCR duplicate removal using the Picard Tools module, MarkDuplicates:
  (a) java -jar bin/MarkDuplicates.jar VALIDATION_STRINGENCY=SILENT I=alignments/NA12878.ALN.sorted.bam O=alignments/NA12878.ALN.dups_removed.bam REMOVE_DUPLICATES=true M=alignments/metrics
(4) Read Group added to alignment files using the Picard Tools module, AddOrReplaceReadGroups:
  (a) java -jar bin/AddOrReplaceReadGroups.jar VALIDATION_STRINGENCY=SILENT I=alignments/NA12878.ALN.dups_removed.bam O=alignments/NA12878.ALN.RG.bam SO=coordinate RGID=NA12878 RGLB=NA12878 RGPL=illumina RGPU=NA12878 RGSM=NA12878 CREATE_INDEX=true
(5) Realignment around indels using the GATK modules RealignerTargetCreator and IndelRealigner:
  (a) java -XX:-DoEscapeAnalysis -jar bin/GenomeAnalysisTK.jar -T RealignerTargetCreator -R genome/hg19.fa -I alignments/NA12878.ALN.RG.bam -known genome/mills.vcf -o tmp/ALN.intervals
  (b) java -XX:-DoEscapeAnalysis -jar bin/GenomeAnalysisTK.jar -T IndelRealigner -R genome/hg19.fa -I alignments/NA12878.ALN.RG.bam -known genome/mills.vcf -o alignments/NA12878.ALN.indels.bam –maxReadsForRealignment 100000 –maxReadsInMemory 1000000 -targetIntervals tmp/ALN.intervals
(6) Base recalibration using the GATK modules BaseRecalibrator and PrintReads:
  (a) java -XX:-DoEscapeAnalysis -jar bin/GenomeAnalysisTK.jar -T BaseRecalibrator -R genome/hg19.fa -I alignments/NA12878.ALN.indels.bam -knownSitesgenome/dbsnp_137.hg19.excluding_sites_after_129.only_standard_chroms.vcf -o tmp/NA12878.ALN.grp
  (b) java -XX:-DoEscapeAnalysis -jar bin/GenomeAnalysisTK.jar -T PrintReads -R genome/hg19.fa -I alignments/NA12878.ALN.indels.bam -BQSR tmp/NA12878.ALN.grp -o alignments/NA12878.ALN.BQSR.bam

### 2.7. Variant calling

Default arguments were used for each variant caller unless it contained a “threads” or “parallel” flag in which case that was used as well. Additionally, indels were called separately from SNVs where possible. Specifically, the commands used are as follows.

#### 2.7.1. FreeBayes

1. freebayes -f genome/hg19.fa -i -X -u -v vcf_files/NA12878.ALIGNER.freebayes.raw.snv.vcf alignments/NA12878.ALIGNER.BQSR.bam
2. freebayes -f genome/hg19.fa -I -X -u -v vcf_files/NA12878.ALIGNER.freebayes.raw.indel.vcf alignments/NA12878.ALIGNER.BQSR.bam

#### 2.7.2. GATK HaplotypeCaller

1. java -XX:-DoEscapeAnalysis -jar bin/GenomeAnalysisTK.jar -T HaplotypeCaller -R genome/hg19.fa -I alignments/NA12878.ALIGNER.BQSR.bam –dbsnp $DBSNP -o vcf_files/NA12878.ALIGNER.HC.raw.vcf -stand_call_conf 50

#### 2.7.3. GATK UnifiedGenotyper

1. java -XX:-DoEscapeAnalysis -jar bin/GenomeAnalysisTK.jar -T UnifiedGenotyper -R genome/hg19.fa -nt 10 -I alignments/NA12878.ALIGNER.BQSR.bam -o vcf_files/NA12878.ALIGNER.UG.raw.snv.vcf -glm SNP -D $DBSNP
2. java -XX:-DoEscapeAnalysis -jar bin/GenomeAnalysisTK.jar -T UnifiedGenotyper -R genome/hg19.fa -nt 10 -I alignments/NA12878.ALIGNER.BQSR.bam -o vcf_files/NA12878.ALIGNER.UG.raw.indel.vcf -glm INDEL -D $MILLS

#### 2.7.4. SAMtools Mpileup

1. samtools mpileup -uf genome/hg19.faalignments/NA12878.ALIGNER.BQSR.bam  |  bcftools view -bvcg - > vcf_files/NA12878.ALIGNER.mpileup.bcf  &&  bcftools view vcf_files/NA12878.ALIGNER.mpileup.bcf  >  vcf_files/NA12878.ALIGNER.mpileup.raw.vcf

#### 2.7.5. SNPSVM

1. java -XX:ParallelGCThreads=10 -jar tools/SNPSVM/snpsvm.jar predict -R genome/hg19.fa -B alignments/NA12878.ALIGNER.BQSR.bam -M tools/SNPSVM/models/default.model -V vcf_files/NA12878.ALIGNER.SNPSVM.raw.vcf

Due to the nonexistence of requisite CIGAR flags in the alignment file, SNPSVM failed to call variants for CUSHAW3, and SAMtools mpileup could not call variants on MOSAIK alignments for the same reason. Also, due to the fact that SNPSVM only detects SNVs, no indels were reported for this program.

### 2.8. Variant Filtration

Filtration varied depending on the variant caller being used. In the cases of GATK HaplotypeCaller and GATK UnifiedGenotyper, the GATK modules, VariantRecalibrator and ApplyRecalibration, were used to filter SNVs using HapMap 3.3, the Omni 2.5 SNP BeadChip, and dbSNP 137 without 1000 Genome data as training sets. For SNPSVM, QUAL scores ≥ 4 and DP values ≥ 6 were used. For FreeBayes and SAMtools, QUAL scores ≥ 20 and DP values ≥ 6 were used.

### 2.9. Variant Comparison

For variant comparison, USeq 8.8.1 was used to compare SNVs shared between all datasets. To compare indels, the vcflib tool vcfintersect was used. The TruSeq hg19 exome bed file truseq_exome_targeted_regions.hg19.bed.chr, obtained in December 11, 2013, was used to restrict comparisons to locations that could be captured by the exome pull down kit used in the sequencing of SRR098401. This file can be obtained from Illumina here: http://support.illumina.com/sequencing/sequencing_kits/truseq_exome_enrichment_kit/downloads.ilmn. To ensure that variants were represented identically between different call sets, the vcflib tool vcfallelicprimitives was used to preprocess vcf files.

### 2.10. Statistical Calculations

*True Positive (TP)*. It is a mutation that was detected by the pipeline being tested and is one that exists in the NIST-GiaB list.

*False Positive (FP)*. It is a mutation that was detected by the pipeline being tested but is one that does not exist in the NIST-GiaB list. 

*True Negative (TN)*. It is a mutation that was not detected by the pipeline being tested and is one that does not exist in the NIST-GiaB list. 

*False Negative (FN)*. It is a mutation that was not detected by the pipeline being tested but is one that does exist in the NIST-GiaB list:

(1) $$\begin{array}{c} \text{PPV}=\frac{\text{TP}}{\left(\text{TP}+\text{FP}\right)},\\ \text{Sensitivity}=\frac{\text{TP}}{\left(\text{TP}+\text{FN}\right)}. \end{array}$$

## 3. Results and Discussion

### 3.1. Prefiltering Variants

When performing variant analysis, one of the many pitfalls that must be taken into consideration is the exome sequence space (as defined by the exome capture kit) and how it can affect the analysis results. In this case, we had a single exome (SRR098401) that was extracted using the Illumina TruSeq exome kit and sequenced on a HiSeq 2000. With this in mind, we wanted to make sure that we were measuring the ability of the bioinformatic tools to do their jobs and not how well the Illumina TruSeq exome capture kit worked. That is, we only want to try to call variants that are supposed to be present in the exons as defined by the pull down kit. For this reason, we use the bed file provided by Illumina, not a generic annotation bed file, for example, RefSeq for hg19. We found that for this particular individual, according to the NIST-GiaB list, there should be a total of 34,886 SNVs and 1,473 indels within the regions defined by the TruSeq bed file.

Once we filtered out variants that were not located in the regions defined by the Illumina TruSeq exome bed file, we went from hundreds of thousands of putative variants (data not shown) to, on average, about 23,000 variants (SNVs and indels) per pipeline (Table 3). This is an important step for researchers to begin with, as it significantly reduces the search space for potentially interesting variants.

### 3.2. Raw Variant Results Compared to GiaB

One aspect we wanted to understand when doing this comparison was the importance of filtering variants detected by these tools. The reason for this is that ideally one would like to have as high a level of sensitivity as possible so that the mutations of interest do not get lost in the filtering process. It therefore behooves us to determine whether or not this step is necessary and to what degree it is necessary, since it is clear from the NIST-GiaB results and the Bamshad et al. [13] review that sensitivity could be an issue.

As we can see in Table 3, filtering is needed more for some variant callers than for others when it comes to SNVs, and it is absolutely necessary for indels. In most cases, the number of TP variants is close to or higher than our expected number of about 20,000 [13], but, on the other hand, in some cases the number of FPs is very high.

Clearly there is a lot of variation in the numbers generated by each pipeline. However, one can find some commonalities in the numbers that likely stem from the algorithmic origins of each tool. FreeBayes produces both the largest number of unfiltered variants and the highest number of FPs. It is likely that we only see this kind of performance from this tool due to the fact that while it is not the only variant caller based on Bayesian inference it is unique in its interpretation of alignments. That is, it is a haplotype-based caller that identifies variants based on the sequence of the reads themselves instead of the alignment, the latter of which is how GATK's UnifiedGenotyper operates.

Additionally we see the Burrows-Wheeler based aligners perform very similarly to each other: Bowtie2, BWA mem, and BWA sampe achieve similar results across the board. One might surmise that this is likely due to the fact that all of these tools utilize similar algorithms when performing their designated task. This observation is supported by the fact that MOSAIK (gapped alignments using the Smith-Waterman algorithm) and CUSHAW3 (a hybrid seeding approach) both have very different underlying algorithms and subsequently produce very different results.

This difference in results correlating with different algorithms is seen best in the SNPSVM results. Of the variant callers, it is the only one that utilizes support vector machines and model building to generate SNV calls. It would appear that while it has the disadvantage of not being as sensitive as other methods it does benefit from being extremely accurate regardless of the aligner being used. This suggests that one is able to skip the filtering step altogether when using this variant caller.

With regard to indels, no aligner seems to stand out among the rest as one that handles this type of mutation well. In fact, when looking at the number of FPs, it is clear that it is the variant caller that plays the largest role in the accuracy of indel identification. Additionally, there are data for neither CUSHAW3 plus SNPSVM nor MOSAIK plus SAMtools mpileup pipelines due to the alignment files not containing the necessary CIGAR strings for the variant callers to function downstream. Lastly, the reason there are no indel data for SNPSVM is because this tool is solely used for identification of SNVs.

### 3.3. Filtered Results Compared to GiaB

As in Table 4, standard filtering practices manage to remove a large number of FP SNVs for each pipeline; however it seems that these filters are a bit too aggressive in most cases for SNV detection, but not strict enough for indels. This is made obvious when looking at the differences in the number of FPs reported in each dataset. For example, Bowtie2 with Freebayes sees a removal of 72,570 FP SNVs (a reduction of 98%) but only a removal of 1,736 FP indels (a reduction of 70%). It should also be noted that the filters used were pipeline-dependent and, for the most part, within each pipeline produced similar reductions in SNV and indel FPs. The one exception here is the number of variants identified from the CUSHAW3 alignments when compared to other alignments: overall the number of TP SNVs is lower, the number of FP SNVs is higher, and it is the only aligner that produces more than 1,000 FP indels after filtering.

Given the fact that filtering significantly reduces the number of TP variants, it might be wise to, with the exception of pipelines using CUSHAW3 and FreeBayes, skip this step when searching for rare, high-impact variants. Instead, one might spend more time on a filtering process that is based on biology rather than statistics. For example, it may make more sense to invest time identifying a small list of variants that are likely to be high-impact: splice site mutations, indels that cause frameshifts, truncation mutations, stop-loss mutations, or mutations in genes that are known to be biologically relevant to the phenotype of interest.

### 3.4. Average TPR and Sensitivity

As can be seen in Table 5, the Positive Predictive Value (PPV) for each tool, with the exception of CUSHAW3, ranges from 91% to 99.9% for SNVs, but the average sensitivity is very low (around 50%). This discrepancy could be due to a number of reasons, but the most likely one is variable depth of coverage across exons. We can see that, in addition to low SNV sensitivity, indel sensitivity is low (around 30%); however the PPV for indels is considerably lower (35.86% to 52.95%). This could be due to any of the following reasons: very short indels are hard to detect by conventional NGS [31], the representation of indels by different variant callers can cause tools to incorrectly claim that two indels are different, or alignment tools produce different representations of the same indel [7].

Perhaps the most likely explanation for both types of mutations is the issue of depth. As is the case with any variant analysis study, an increase in depth of coverage leads to an increase in sensitivity, but it is impossible to guarantee good depth of coverage due to the inability of exome capture kits to uniformly pull down exons [32–34]. Additionally, no single exome capture kit covers every exon. Indeed, it has been shown that variant analysis of whole genome sequencing at an average depth lower than an exome performs better due to the uniformity of said depth. Thus, it is likely that a large number of variants are missing due to the fact that the NIST-GiaB list was created from a compilation of exomic and genomic sequencing data. Ultimately, to achieve proper sensitivity one will eventually need to perform whole genome sequencing, but that is currently cost-prohibitive for most labs. Fortunately, this cost is continuing to drop, and we will soon see a gradual shift from exome analysis to the more complete whole genome analysis.

### 3.5. Sensitivity as a Function of Depth

**Figure 2:**
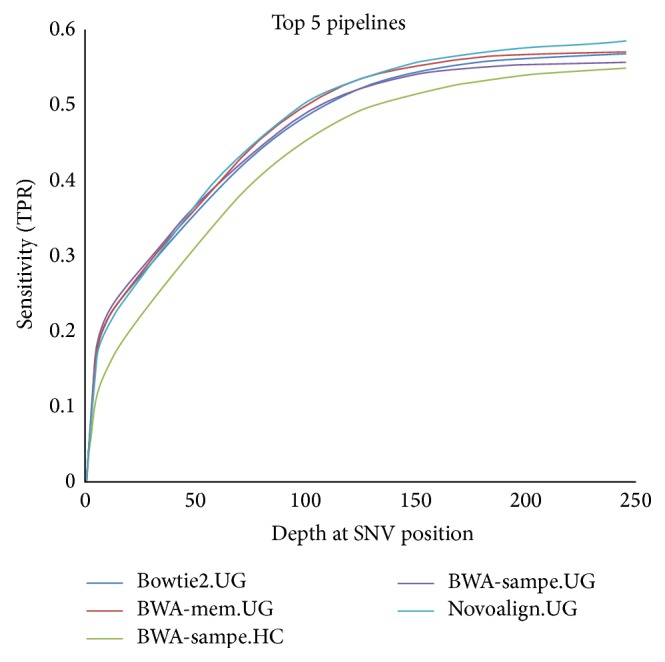
Sensitivity as a function of depth for the top five pipelines. The top five pipelines are shown here with the depth of every SNV plotted against sensitivity.

**Figure 3:**
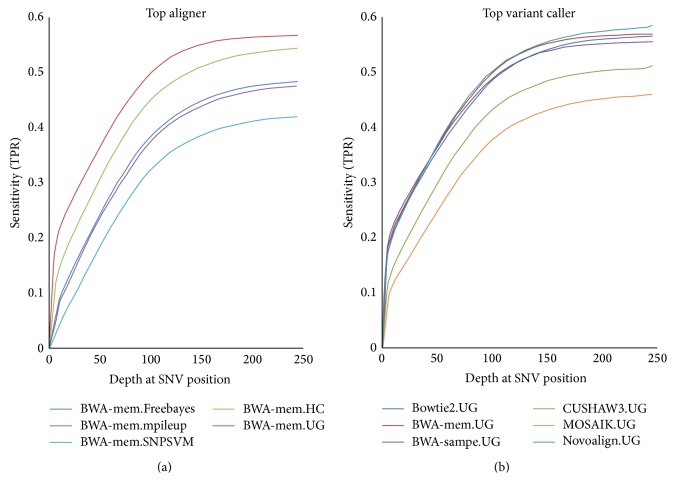
Sensitivity as a function of depth for the top aligner and top variant caller. (a) Results for the depth of every SNV plotted against sensitivity for the top aligner, BWA mem, paired with every variant caller. (b) Results for the depth of every SNV plotted against sensitivity for the top variant caller, GATK UnifiedGenotyper, paired with every aligner.

Because sensitivity reflects one of the most important performance metrics of a tool and most of the tools struggle to achieve sensitivity higher than 50%, we would like to further explore how depth affected variant calling sensitivity. We looked at a number of different combinations of tools to determine what the best pipelines, variant callers, and aligners were. For Figure 2, we took the five best combinations of variant callers and aligners as determined by their sensitivity and false positive rate (FPR). That is, we selected those which had the highest number of TP SNVs called in addition to the lowest number of FP SNVs. Upon inspection, the thing that stands out immediately is that the sensitivity is lower than expected. All of the pipelines perform at roughly the same level: they identify most of their variants by the time a depth of about 150x has been reached, which indicates that this depth is likely sufficient and that the number of missing variants is probably due to certain exons having lower than average coverage. Note that four out of the five best performing pipelines have GATK UnifiedGenotyper as their variant caller, demonstrating its superior performance irrespective of the aligner used as shown in Figure 3(b).

In addition to looking at the top five pipelines, we determined it would be useful to perform the same analysis on the best aligner coupled with every variant caller (Figure 3(a)), as well as the best variant caller coupled with every aligner (Figure 3(b)). As with the pipelines, the best aligner was identified as that which produced the highest number of TP SNVs and the lowest number of FP SNVs—in this case BWA mem. Despite having the best alignment to work with, we still see a fairly large difference between the variant callers, which is likely attributable to the different algorithms they employ (Figure 3(a)). However, in the case of the best performing variant caller, GATK UnifiedGenotyper, there seems to be less variation among the top four aligners indicating that it performs fairly well in most situations with the exceptions being CUSAHW3 and MOSAIK.

### 3.6. Shared Variants among the Top Pipelines

**Figure 4:**
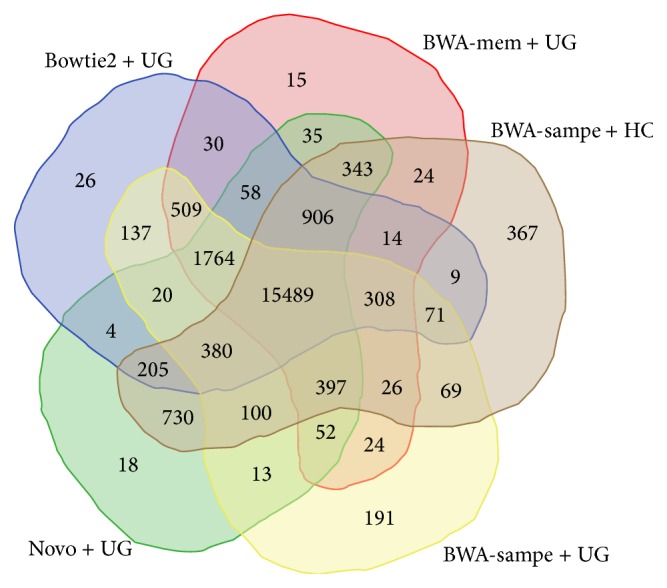
The intersection of the SNVs identified by the top five pipelines.

Lastly, we wanted to know just how unique the variant call sets were between the different pipelines. To do this, we again focused on the top five variant calling pipelines: Bowtie2 plus UnifiedGenotyper, BWA mem plus UnifiedGenotyper, BWA sampe plus HaplotypeCaller, BWA sampe plus UnifiedGenotyper, and Novoalign plus UnifiedGenotyper. As can be seen in Figure 4, there is a large amount of overlap between the five different pipelines in question, with 15,489 SNVs (70%) shared out of a total of 22,324 distinct variants. However, one could also argue that this is largely due to four of the five pipelines using the UnifiedGenotyper as their variant caller. This notion is corroborated by the fact that the largest number of variants unique to a pipeline, 367, belongs to the BWA sampe plus HaplotypeCaller combination. It is also worth noting that the second highest number of unique SNVs also belongs to the BWA sampe aligner, so it is possible that the high number of unique SNVs is better attributed to the aligner than the variant caller.

## 4. Conclusions

We found that among the thirty different pipelines tested Novoalign plus GATK UnifiedGenotyper exhibited the highest sensitivity while maintaining a low number of FP for SNVs. Of the aligners, BWA mem consistently performed the best, but results still varied greatly depending on the variant caller used. Naturally, it follows that the best variant caller, GATK UnifiedGenotyper, mostly produced similar results regardless of the aligner used. However, it is readily apparent that indels are still difficult for any pipeline to handle with none of the pipelines achieving an average sensitivity higher than 33% or a PPV higher than 53%. In addition to the low overall performance we see in detecting indels, sensitivity, regardless of mutation type, is a problem for every pipeline outlined in this paper. The expected number of SNVs for NA12878's exome is 34,886, but even when using the union of all the variants identified by the top five pipelines, the greatest number identified was very low (22,324). It seems that while still very useful exome analysis has its limitations even when it comes to something as seemingly simple as SNV detection.

## Figures and Tables

**Table 1:** Alignment percentages for filtered reads and unfiltered reads. The average depth of coverage is for the alignment files created with the filtered reads.

Aligner	% reads aligned (unfiltered)	% reads aligned (filtered)	Average depth of coverage
Bowtie2	89.73	98.73	47.97
BWA mem	92.91	99.85	46.89
BWA sampe	85.95	97.49	46.67
CUSHAW3	85.00	99.81	47.69
MOSAIK	85.68	96.22	45.14
Novoalign	82.21	94.20	45.62

**Table 2:** These are the 11 different tools used that made up the 30 (six aligners ∗ five variant callers) different pipelines. Software versions are also included to ensure reproducibility.

Tool	Type	Version	Reference
Bowtie2	Aligner	2.1.0	[14]
BWA sampe	Aligner	0.7.5a	[15]
BWA mem	Aligner	0.7.5a	[16]
CUSHAW3	Aligner	3.0.3	[17]
MOSAIK	Aligner	2.2.3	[18]
Novoalign	Aligner	3.02.07	N/A
FreeBayes	Genotyper	v9.9.2-19-g011561f	[22]
GATK HaplotypeCaller	Genotyper	2.7-2	N/A
GATK UnifiedGenotyper	Genotyper	2.7-2	[23]
SAMtools mpileup	Genotyper	0.1.19	[24]
SNPSVM	Genotyper	0.01	[25]

**Table 3:** Raw variant statistics for the 30 pipelines, including SNVs and indels.

Aligner	Genotyper	Raw TP SNVs	Raw FP SNVs	Raw TP indels	Raw FP indels
Bowtie2	FreeBayes	23,985	73,473	806	2,482
Bowtie2	GATK HC	21,631	273	771	1,103
Bowtie2	GATK UG	25,136	2,276	418	420
Bowtie2	mpileup	21,930	1,030	734	1,414
Bowtie2	SNPSVM	17,613	47	—	—
BWA mem	FreeBayes	23,857	18,256	785	2,088
BWA mem	GATK HC	21,707	367	779	1,348
BWA mem	GATK UG	21,925	213	402	408
BWA mem	mpileup	25,081	2,129	761	1,772
BWA mem	SNPSVM	17,920	65	—	—
BWA sampe	FreeBayes	23,789	27,143	737	1,872
BWA sampe	GATK HC	21,878	263	758	1,161
BWA sampe	GATK UG	22,153	321	394	385
BWA sampe	mpileup	25,206	2,205	684	1,401
BWA sampe	SNPSVM	18,017	78	—	—
CUSHAW3	FreeBayes	23,191	53,525	624	3,310
CUSHAW3	GATK HC	19,673	14,814	751	4,727
CUSHAW3	GATK UG	19,113	13,184	360	1,005
CUSHAW3	mpileup	22,171	9,694	681	1,983
CUSHAW3	SNPSVM	—	—	—	—
MOSAIK	FreeBayes	23,373	39,203	783	3,359
MOSAIK	GATK HC	13,528	111	500	458
MOSAIK	GATK UG	17,147	76	392	284
MOSAIK	mpileup	—	—	—	—
MOSAIK	SNPSVM	14,586	8	—	—
Novoalign	FreeBayes	22,794	2,970	678	1,554
Novoalign	GATK HC	21,407	473	779	1,370
Novoalign	GATK UG	21,113	144	387	365
Novoalign	mpileup	24,512	1,861	773	1,781
Novoalign	SNPSVM	17,109	164	—	—

**Table 4:** Filtered variant statistics for the 30 pipelines, including SNVs and indels.

Aligner	Genotyper	Filtered TP SNVs	Filtered FP SNVs	Filtered TP indels	Filtered FP indels
Bowtie2	FreeBayes	17,504	903	481	746
Bowtie2	GATK HC	17,330	29	648	687
Bowtie2	GATK UG	19,937	49	395	338
Bowtie2	mpileup	17,049	153	402	541
Bowtie2	SNPSVM	13,983	8	—	—
BWA mem	FreeBayes	17,376	347	461	739
BWA mem	GATK HC	19,388	302	689	860
BWA mem	GATK UG	20,000	48	397	355
BWA mem	mpileup	17,070	57	403	606
BWA mem	SNPSVM	15,060	10	—	—
BWA sampe	FreeBayes	17,435	450	443	647
BWA sampe	GATK HC	19,438	214	630	725
BWA sampe	GATK UG	19,557	27	384	336
BWA sampe	mpileup	17,049	111	387	518
BWA sampe	SNPSVM	15,218	10	—	
CUSHAW3	FreeBayes	16,620	7,627	362	1,294
CUSHAW3	GATK HC	16,590	2,195	665	1,551
CUSHAW3	GATK UG	17,939	2,202	357	545
CUSHAW3	mpileup	15,942	4,029	368	796
CUSHAW3	SNPSVM	—	—	—	—
MOSAIK	FreeBayes	17,177	679	458	645
MOSAIK	GATK HC	11,616	33	426	255
MOSAIK	GATK UG	16,423	42	381	224
MOSAIK	mpileup	—	—	—	—
MOSAIK	SNPSVM	4,727	3	—	—
Novoalign	FreeBayes	16,658	219	384	559
Novoalign	GATK HC	19,406	385	702	872
Novoalign	GATK UG	20,521	46	386	315
Novoalign	mpileup	16,493	62	396	579
Novoalign	SNPSVM	14,451	18	—	—

**Table 5:** Average Positive Predictive Value (PPV) and sensitivity for each tool.

Tool	Average SNV PPV	Average SNV sensitivity	Average indel PPV	Average indel sensitivity
Bowtie2	98.69%	49.19%	45.45%	32.69%
BWA mem	99.15%	50.96%	43.24%	33.10%
BWA sampe	99.09%	50.85%	45.31%	31.30%
CUSHAW3	80.69%	48.08%	29.50%	29.74%
MOSAIK	98.51%	35.79%	52.95%	28.63%
Novoalign	99.17%	50.18%	44.55%	31.70%
FreeBayes	90.95%	51.00%	35.86%	32.79%
GATK HaplotypeCaller	97.05%	51.03%	43.17%	31.79%
GATK UnifiedGenotyper	97.93%	50.77%	52.12%	31.57%
SAMtools mpileup	94.99%	50.76%	39.15%	31.30%
SNPSVM	99.92%	50.85%	N/A	N/A
